# Characterizing and Modeling Transformation-Induced Plasticity in 13Cr-4Ni Welds upon Cooling

**DOI:** 10.3390/ma16227166

**Published:** 2023-11-15

**Authors:** Jean-Benoit Lévesque, Carlo Baillargeon, Daniel Paquet, Jacques Lanteigne, Henri Champliaud

**Affiliations:** 1 Institut de Recherche d’Hydro-Québec, 1800 Boul. Lionel-Boulet, Varennes, QC J3X 1S1, Canada; 2 Department of Mechanical Engineering, École de Technologie Supérieure, 1100 Rue Notre-Dame O, Montréal, QC H3C 1K3, Canada

**Keywords:** martensitic stainless steel, phase transformation, TRIP, dilatometry, hydraulic turbine, welding simulation

## Abstract

Dilatometric experiments were conducted with the main purpose of measuring the transformation-induced coefficients of 13% chromium and 4% nickel, which are martensitic stainless steel base and filler materials used for hydraulic turbine manufacturing. To this end, a set of experiments was conducted in a quenching dilatometer equipped with loading capabilities. The measurement system was further improved by means of modified pushrods to allow for the use of specimens with geometries that are compliant with tensile test standards. This improvement allowed for the measurement of the materials’ phases and respective yield strengths. The dataset was further used to determine the relationship between the applied external stress and the martensitic start temperature ($M_{s}$) upon cooling. The TRIP coefficient’s K values for both the S41500 steel and E410NiMo filler material were measured at $8.12\times 10^{-5}$ and $7.11\times 10^{-5}$, respectively. Additionally, the solid phase transformation model parameters for both the austenitic and martensitic transformation of the filler material were measured. These parameters were then used to model austenitic-phase-transformation kinetics and martensite transformation, including transformation-induced plasticity effects. Good agreement was achieved between the calculation and the experiments.

## 1. Introduction

Hydroelectric turbines commonly fail due to fatigue damage, resulting in unexpected and often long-term shutdowns, productivity losses, and significant expenses for repairs. Francis-type turbine runners are manufactured from large cast parts, with the blades welded to the crown and ring belt. A common base and filler material used for their manufacture is martensitic stainless steel with a nominal content of 13% chromium and 4% nickel. Over the years, multiple incidents have revealed that fatigue cracks typically initiate and grow within or near the fillet weld in the heat-affected zone (HAZ), which is the region of the structure known to experience the highest operation stresses and high residual stress due to welding processes and potentially containing casting and welding defects. Once discovered, these cracks are usually repaired on-site using gouging and welding operations.

It is well known that residual stresses left by manufacturing and repairing operations, such as welding or heat treatment, have an effect on the initiation and propagation of fatigue cracks [1]. For example, Savaria et al. [2] demonstrated that the residual stress field left by induction gear tooth heat treatment had a significant effect on the fatigue life of MS6414 steel. Paquet et al. [3] showed that the evolution of residual stresses after loading could lead, in some cases, to unexpected fatigue life, stating that great care should be taken to consider the phenomenon in fatigue life calculations. Itoh et al. [4] conducted experiments to study the effect of welding residual stresses on fatigue crack propagation rate and found that the effective stress intensity range, $\Delta K_{eff}$, must consider residual stress distribution and related crack opening effects. This has been confirmed by Deschênes et al. [5] on 13Cr-4Ni steel for the use of a specimen specially designed to include welding residual stress while avoiding microstructure alteration. Liljedahl et al. [6] performed in situ neutron diffraction on welding residual stress during fatigue tests to analyze residual stress redistribution and successfully predicted fatigue crack growth. Jones et al. [7], used plastically bent specimens to evaluate superposition concepts for fatigue crack growth rate estimation, concluding that the method is accurate but relies on precise knowledge of the residual stress field. This level of knowledge of residual stress distribution arising from turbine runner assembly operations cannot yet be known experimentally. One way to circumvent this lack of experimental capacity is to model the fabrication steps.

In the last few decades, finite element method frameworks for the simulation of residual stresses left by welding operations have proven effective [8,9]. Among others, the works of Deng et al. [10] and Rikken et al. [11] have shown the importance of considering solid-state phase transformation (SSPT) in the simulation of the welding processes of materials experiencing martensitic transformation upon cooling. Other studies [12,13,14,15] have also highlighted the need to consider transformation-induced plasticity (TRIP) during martensitic transformation in order to compute a reliable stress distribution. These plastic strains are induced as the SSPT progresses while stress, σ, is applied, even if the stress is below the yield strength of the weakest phase. This phenomenon is commonly explained by two mechanisms: (i) a mismatch of the volume expansion coefficient results of microplasticity in the weaker phase [16], and (ii) the application of stress, which promotes the formation of preferred martensite variants [17], resulting in anisotropic permanent strains.

For the purpose of evaluating TRIP strains, Leblond et al. [18,19] proposed an undisputed model. In this model, the total strain rate, $\dot{\epsilon }$, is decomposed as follows:

(1) $$\dot{\epsilon }={\dot{\epsilon }}^{e}+{\dot{\epsilon }}^{thm}+{\dot{\epsilon }}^{p}$$

where ${\dot{\epsilon }}^{e}$ is the elastic strain rate, ${\dot{\epsilon }}^{thm}$ is the thermo-metallurgical strain rate, which includes thermal strains as well as the strains resulting from the evolution of phases in proportion, and ${\dot{\epsilon }}^{p}$ is the plastic strain rate. This last term is a summation of the contributions of the classical plasticity strain rate, which is time-independent in this case, ${\dot{\epsilon }}^{cp}$, and relies on external stress and temperature rates, and the transformation-induced plasticity strain rate, ${\dot{\epsilon }}^{tp}$, which depends on the phase transformation rate. This summation is decomposed as the sum of

(2) $${\dot{\epsilon }}^{p}={\dot{\epsilon }}^{cp}+{\dot{\epsilon }}^{tp}$$

The term ${\dot{\epsilon }}^{tp}$ can be obtained via a relation to the following form:

(3) $${\dot{\epsilon }}^{tp}=\frac{3}{2}KS\phi \left(f\right)\dot{f}$$

where *K* is the TRIP coefficient, S is the stress deviatoric tensor, ϕ(f) is a normalized function of the volume increase in the product phase formed, for instance, expressed as ϕ(f)=f(1−lnf), and $\dot{f}$ is the transformation rate. In their article series, Leblond et al. thoroughly derive each of the terms of Equations (1)–(3) based on material properties and constitutive relations. They even supply a theoretical background for the determination of the TRIP coefficient based on the difference between the expansion curves of the phases and the yield strength of the parent phase. However, the determination of the TRIP coefficient can be conducted experimentally [20,21], which provides an even better evaluation of the final TRIP strains.

This paper proposes the measurement of the TRIP coefficients of both the base and filler 13Cr-4Ni martensitic stainless steels used for turbine runner fabrication and assembly. Attention is also given to the effect of the application of external stress on the martensitic start temperature. Along with the help of the model proposed by Leblond et al., the values measured are then used to evaluate the strains that occur as martensitic transformation takes place and transformation-induced plasticity is triggered by the application of an external load. The results were then compared to the experimental results.

## 2. Materials and Methods

### 2.1. Materials

**Figure 1:**
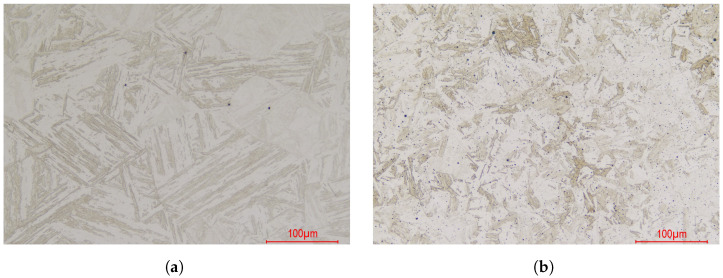
Micrographs of the materials under study: (**a**) UNS S41500 base metal; (**b**) E410NiMo base metal.

The two materials under study are (1) a base metal, 13Cr-4Ni soft martensitic hot-rolled stainless steel, denominated as UNS S41500, as per ASTM-A240/A240M-18 [22], and (2) the filler material, a homogeneous electrode, designated E410NiMo according to ASME BPVC.II.C [23]. The base material used in this study was in the shape of a 60 mm thick plate (ArcelorMittal, Saint-Denis, France). The filler metal (Hobart Welding Products, Appleton, WI, USA) was deposited in several layers on a substrate plate in order to form an 80 mm thick weldment and allow further specimen sampling. Both materials were fully austenitized at 1050${}^{{}^{\circ}}$ for 1 h for microstructure homogenization and then furnace-cooled to room temperature in order to obtain fully martensitic microstructures. The micrographs of both materials, obtained at a 200× magnification, are presented in Figure 1. UNS S41500 shows a fully martensitic microstructure with few solid inclusions located at the prior austenitic grains boundaries. The E410NiMo filler material also presents a fully martensitic microstructure, with smaller prior austenitic grains and smaller martensite blocs. Many micrometric inclusions of flux and the microporosities left by the welding operations can be observed.

An analysis of the chemical content was conducted on both materials to ensure compliance with the standards. The results, provided in weight percentage (wt%), are shown in Table 1. The chemical contents were measured by glow discharge atomic emission spectrometry, except for carbon and phosphorus, which were measured by using the mean of the combustion infra-red detection technique.

**Figure 2:**
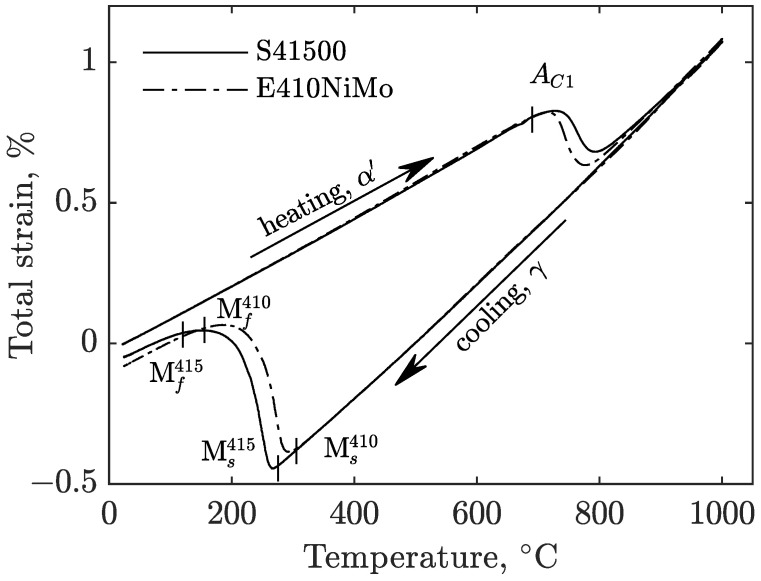
Dilatation as a function of temperature during the heating and cooling of S41500 and E410NiMo soft martensitic stainless steels.

Figure 2 shows the results of the dilatometric experiments carried out in the preamble of this study. It shows that both materials follow nearly the same thermal expansion in both the fully austenitic and martensitic states. However, significant differences arise when focusing on the regions where solid phase transformations occurred. During heating at the same rate, austenitic transformation starts at the same temperature but behaves differently as austenite grows more quickly in the E410NiMo filler material. Upon cooling, the temperature at which martensitic transformation starts also differs. A martensitic transformation start temperature of E410NiMo, $M_{s}^{410}$, was measured at $307.9\;\pm \;8.4{\;}^{{}^{\circ}}$C, which is noticeably higher than that of S41500, $M_{s}^{415}$, at $261.7\;\pm \;7.0{\;}^{{}^{\circ}}$C. This trend was also observed for the end of the martensitic transformation temperatures, with $M_{f}^{410}$ measured at $161.1\;\pm \;19.6{\;}^{{}^{\circ}}$C and $M_{f}^{415}$ measured at $127.2\;\pm \;8.1{\;}^{{}^{\circ}}$C.

### 2.2. Experimental Setup

**Figure 3:**
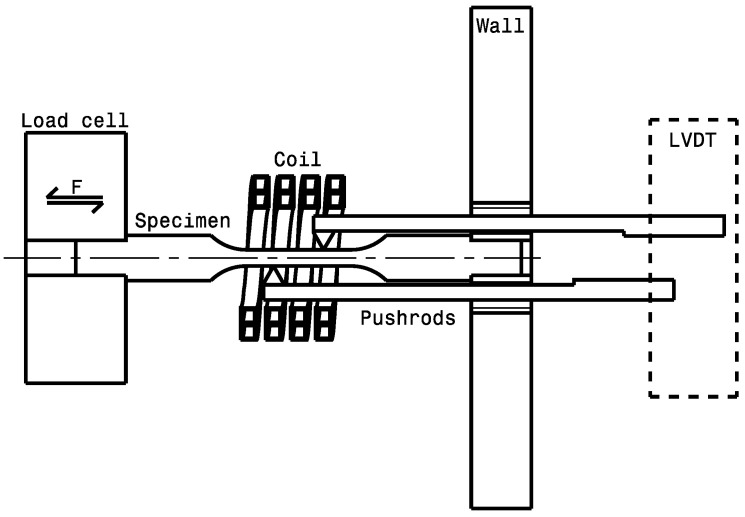
Schematic of the test chamber of the quenching dilatometer. The force, F, compressive or tensile, is applied to the sample through the Load cell. The other end of the sample is fixed to the wall.

A set of experiments was designed for both materials. These experiments needed to allow for a measurement of material strain while changing temperature. The apparatus also needed to permit a controlled tensile or compressive stress to be applied to the specimen in order to trigger transformation-induced plasticity upon cooling the specimen. The device used for this purpose was a DIL805 A/D/T™ quenching dilatometer (TA Instruments, New Castle, DE, USA). The quenching dilatometer was set to the tension mode, which enables the use of a hydraulic actuator to apply both tensile and compressive stresses. The actuator was linked to the sample through a load cell, allowing for the measurement of the applied force, F. Heating of the samples was carried out by using an induction coil, well-known for its fast heating capability. Cooling was controlled by the admission of an argon flow around the sample. Strain measurements were accomplished by using a linear variable differential transformer (LVDT). In this study, an improvement to the measuring system is proposed in order to allow for the use of a specimen geometry that complies with the ASTM E8/E8M [24] standard for tensile tests. A modification of the extensions of the LVDT, namely push-rods, was made to introduce wedges pointing toward the specimen’s reduced section. A small force, radial to the specimen, was applied on the wedge during the installation and was maintained during the test. The movement associated with the specimen strain was then followed as the wedge’s tip stayed on the sample due to friction. Prior to mounting a specimen, the LVDT must be tared to the initial gauge length, with a calibration specimen consisting of a tensile sample with 2 radial grooves precisely distanced by 10.0 mm. The use of this configuration removes the stress concentration in the reduced section of the usual geometry and thereby prevents the formation of plastic strain in the early loading stages, which, in turn, enables the measurement of stress-strain curves with small-size specimens at different temperatures. Figure 3 shows a schematic of the system used for the experiments.

### 2.3. Specimen Design

The specimen is designed to meet the ASTM E8/E8M [24] geometric requirements for tensile tests on a cylindrical specimen (testing device loading attachment geometry and capacity). The maximum load that can be applied by the actuator is 10 kN, and the room temperature ultimate tensile stress of the steels under study falls just below 1200 MPa. Therefore, a reduced section with a 3.25 mm nominal diameter was selected, allowing the materials to reach their ultimate strength during a tensile test. The length of the reduced section is 21 mm, with a fillet radius of 10.0 mm. A finite element simulation was performed to validate that no buckling would occur at the compression stress level in this study. Specimen fabrication was accomplished by using conventional machining, with the specimen axis oriented along the rolling direction of the S41500 steel and along the welding direction for the E410NiMo deposit.

### 2.4. Thermal Cycle

**Figure 4:**
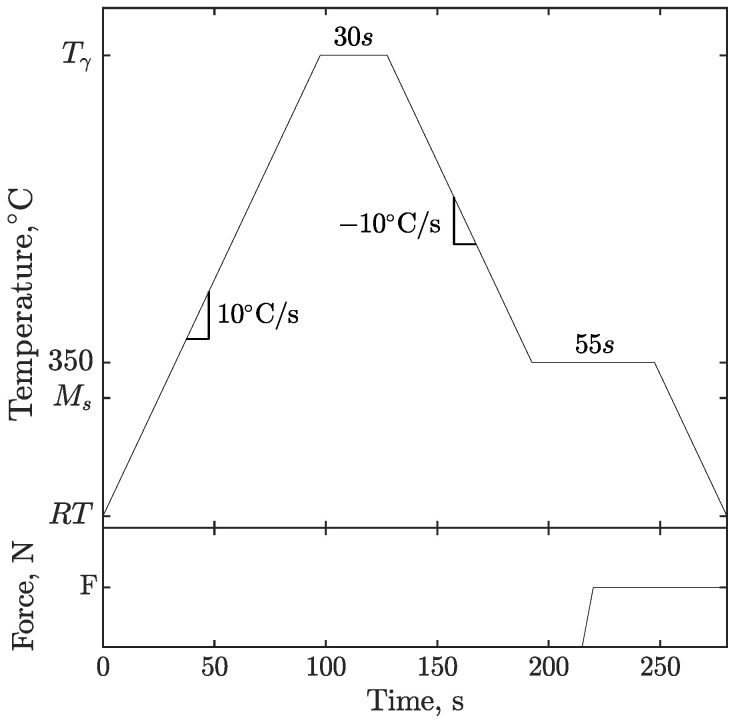
TRIP experiments: thermal and loading history.

Since one of the goals of this work was to determine the materials’ transformation-induced plasticity parameters during martensitic transformation, the temperature of the specimen needed to be modulated for SSPT to occur. The thermal history is shown in Figure 4 and described here. The specimen was heated to a sufficient temperature to allow the martensite-to-austenite transformation to be completed. From room temperature, RT, the specimen was heated at a continuous heating rate of $10{\;}^{{}^{\circ}}$C/s until it reached an austenitization temperature, $T_{\gamma }$, of $1000{\;}^{{}^{\circ}}$C. Once this temperature was attained, it was maintained for a 30-s soaking time before starting the cooling process. This short soaking time ensured the complete austenitization of the specimen’s reduced section and conformed to the thermal histories encountered during the welding process. From the thermal finite element method simulation of welding performed in previous work [25], it is known that the cooling rate that occurs in the weld, following the flux-cored arc welding (FCAW) process, is around $10{\;}^{{}^{\circ}}$C/s as martensitic transformation starts. Therefore, this cooling rate was maintained from $T_{\gamma }$ until the specimen temperature dropped to $350{\;}^{{}^{\circ}}$C, a temperature slightly above martensitic transformation, which is the $M_{s}$ of the studied steels. Both materials are known to experience martensitic transformation independently of the cooling rate, allowing the temperature to be held just before the martensitic transformation starts. A 55-s dwell time was maintained while a load was gradually applied to the specimen. The cooling was then resumed at the same previous rate and continued until RT was reached, allowing martensitic transformation to be completed.

### 2.5. Loading for TRIP Experiments

The amount of TRIP strain encountered during transformation is related to the deviatoric stress tensor and the yield stress of the parent phase. Multiple stress levels must be performed to determine the materials’ transformation-induced plasticity parameters. These stress levels must be lower than the parent austenitic phase yield stress, $\sigma_{\gamma }^{y}$, to prevent the plasticity of the parent phase prior to phase transformation. The yield stress of the austenitic phase was measured using the previously described apparatus and specimens for both materials. The temperature at which to perform the tensile test has to be higher than the martensitic start temperature, $M_{s}$, which was measured at $307.9{\;}^{{}^{\circ}}$C for E410NiMo, as stated in Section 2.1. Hence, a temperature of $350{\;}^{{}^{\circ}}$C was chosen to perform the tensile tests after a full austenitization at $1000{\;}^{{}^{\circ}}$C. The tensile tests were performed at a constant loading rate of 10 N/s. According to these tests, yield strengths of 90 MPa and 110 MPa were measured for S41500 and E410NiMo, respectively.

The tested stress levels were then determined from these values. Stress levels of the same magnitude in tension and compression were tested. This strategy allows for the transformation-induced strain to be isolated. As suggested by Neubert et al. [20], by averaging the absolute value of the final total strains of the paired stress levels, the strains due to phase thermal coefficient mismatches and prestresses vanished. Table 2 summarizes the stress levels tested for both materials. Two specimens were tested for each stress level. The bottom of Figure 4 shows the loading history over time. The magnitude of the force was determined prior to the experiment in order to obtain the intended stress value. This force was gradually applied during the $350{\;}^{{}^{\circ}}$C isotherm in a 5 s time lapse before sample cooling was resumed.

## 3. Results and Discussion

### 3.1. Effect of the Application of Stress on the Total Strain upon Martensitic Transformation

**Figure 5:**
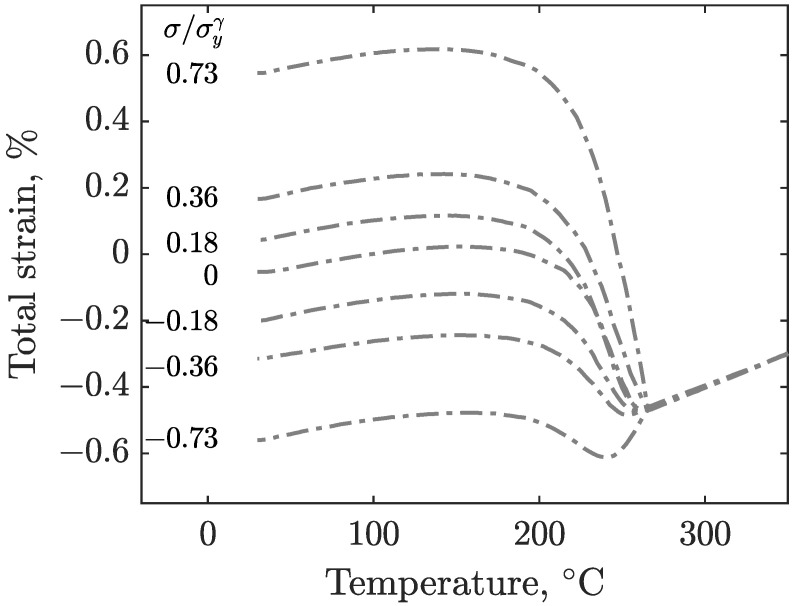
Total strain measured during the cooling of the S41500 specimens for various magnitudes of uniaxial stress (compressive and tensile).

**Figure 6:**
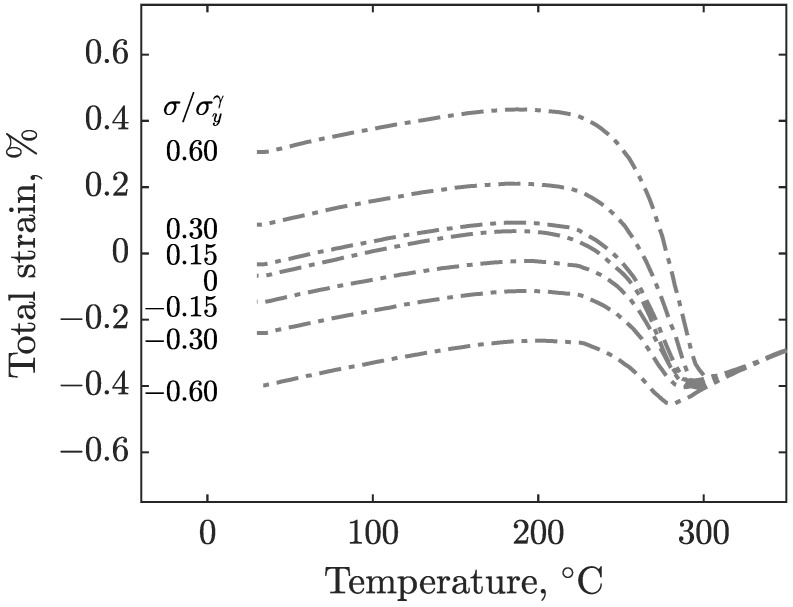
Total strain measured during the cooling of the E410NiMo specimens for various magnitudes of uniaxial stress (compressive and tensile).

The results of the experiments described in Section 2 are shown in Figure 5 for S41500, and Figure 6 shows the results of E410NiMo. These figures show the evolution of strain as the specimens are cooled from the $350{\;}^{{}^{\circ}}$C isotherm after the imposition of the load. The corresponding load-to-yield stress ratios are given in the inset. The strain signals are filtered by means of a moving average to remove noise. Since the obtained data are similar for a given stress level, the test results of both specimens are averaged for each load level for the sake of clarity. At first, it can be observed that the final total strains are distributed roughly linearly with applied stress. The effect of applied stress on $M_{s}$ can also be seen in these figures. With increasing stress magnitude for both tension and compression, the martensitic transformation starts at higher temperatures.

### 3.2. Experimental Determination of the TRIP Parameter

**Figure 7:**
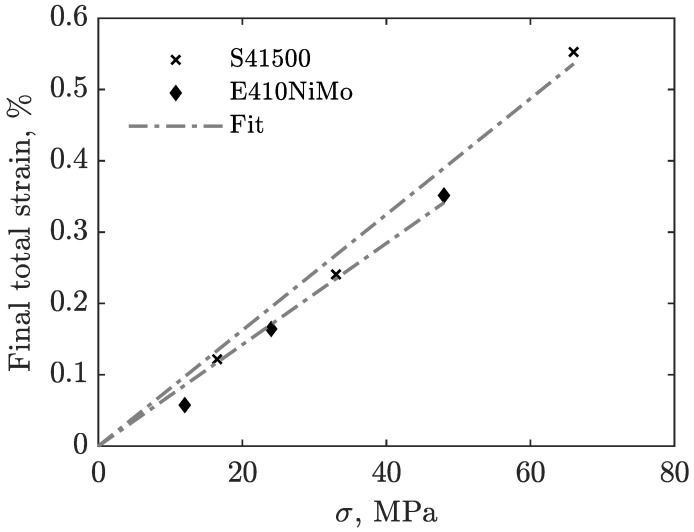
Final transformation-induced strains as a function of the applied stress magnitude for S41500 and E410NiMo.

From these experimental results, and with the assumption that the TRIP strain rate is a function of the transformation rate $\dot{f}$, the TRIP parameter *K* can be determined, as indicated in (3). Figure 7 shows the final transformation-induced strains as a function of the magnitude of the applied stress for both materials. The absolute final strain, which is the strain left at the end of the experiment when reaching room temperature, is used for this purpose. During the tests, other sources, such as prestress from specimen preparation or internal thermal forces, contribute to plastic strain. However, it is assumed that these remain constant between the experiments. Therefore, the TRIP strain for a given stress magnitude can be obtained by calculating the difference between the final strain of the tests with and without applied stress. Then, the results of the experiments with the same equivalent stress, both in tension and compression, are averaged. This procedure reveals the amount of strain attributable to the transformation-induced plasticity as the other plastic strain sources vanish. The stress-strain couples are used to perform a linear regression, assuming that TRIP strain is zero when no stress is applied. The slope of this regression is the transformation-induced plasticity parameter.

The values obtained are $K^{415}=8.12\times 10^{-5}$ and $K^{410}=7.11\times 10^{-5}$ for both materials. The TRIP parameter for S41500 steel is slightly higher than that for E410NiMo, which complies with their respective measured yield stress. As stated in Section 2, the S41500 austenitic phase yield stress is lower than that of E410NiMo, which means that for an equivalent external stress ratio, the austenitic parent phase of S41500 stainless steel is more likely to develop microplastic strains as the harder martensitic phase grows, resulting in a volumetric expansion. The TRIP parameter value obtained for S41500 steel in the current study is in accordance with the $7.7\times 10^{-5}$ value obtained in the preamble of this work, where the tests were performed on a tensile test frame equipped with an electric furnace and a high-temperature extensometer. The value obtained for E410NiMo also compares well with the work of Neubert et al. [20], who obtained $5.3\times 10^{-5}$ using another type of equipment and a slightly different material.

### 3.3. Relationship between $M_{s}$ and Applied Stress

**Figure 8:**
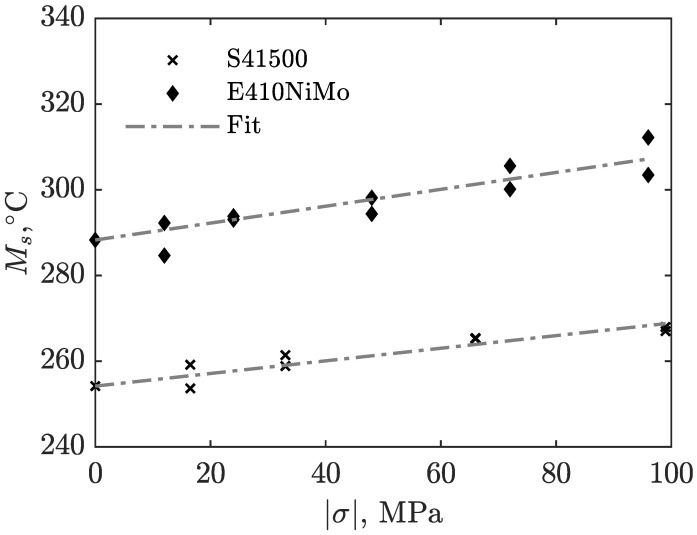
Martensitic start transformation as a function of applied stress.

In addition to the TRIP parameters, the experimental results, along with the results of some higher stress ratios not suitable for TRIP parameters measurements, were used further to determine a relationship between the applied external stress and the martensitic transformation start temperature. The temperature and strain measurements are processed in the same manner as in previous work [25]. Polynomial regressions were performed on the strain recording: one on the part before and another on the part after the martensitic transformation had started. The point where the extrapolated regression curves cross each other is then referenced as the theoretical martensite start temperature, $T_{KM}$. This process is repeated on each set of measurements to obtain scattered data on the effect of applied stress on the martensitic transformation of both materials at distinct plots. The regressions are constrained to reduce to the measured $T_{KM}$ with respect to the material when no stress was applied, which was measured as being $254.6\;\pm \;4.2{\;}^{{}^{\circ}}$C and $288.3\pm 3.6{\;}^{{}^{\circ}}$C for S41500 and E410NiMo, respectively. The results are shown in Figure 8, along with a linear regression of those data points. The coefficients of determination, R${}^{2}$, are 0.875 and 0.836 for S41500 and E410NiMo, respectively, with S41500 steel showing better agreement. This might be because E410NiMo is a weld metal and, therefore, shows a columnar microstructure more prone to heterogeneity. Equations (4) and (5) give the above-mentioned relations for each material.

(4) $$T_{KM}^{415}=254.6+0.147\left|\sigma \right|$$

(5) $$T_{KM}^{410}=288.3+0.197\left|\sigma \right|$$

### 3.4. Validation of the Transformation-Induced Plasticity Model

The transformation-induced plasticity parameters calculated in Section 3.2 were used, along with the martensite start temperature equations defined in Section 3.3, to model the materials’ behavior under the experimental conditions described in Section 2. For this purpose, the evolution of the phase proportion must be modeled as temperature and time vary. The S41500 stainless steel material parameters for modeling phase transformation during heating and cooling were experimentally determined [25].

The austenitic transformation was modeled using the so-called Johnson–Mehl–Avrami–Kolmogorov (JMAK) model [26,27,28,29] by considering the isochronal heating of dilatometric specimens. The same methodology was used here to characterize the parameters for the E410NiMo filler material, which, in turn, allowed us to determine the activation energy ($E_{a}$) by using the Kissinger method [30], the chemical reaction pre-exponential factor of the Arrhenius function, $k_{0}$, and the JMAK exponent, *n*. These parameters allow the next equations to be used to model the proportion of the austenite phase during austenitic transformation:

(6) $$f_{\gamma }=1-e^{-{\left(k_{0}s\right)}^{n}}$$

with

(7) $$s=\sum_{i=1}^{m}e^{\frac{-E_{a}}{RT_{i}}}\Delta t_{i}$$

where $\Delta t_{i}$ and $T_{i}$ are the ith intervals of time and temperature, respectively, *m* is the total number of increments, and *R* is the universal gas constant.

The martensitic transformation was modeled by using a Koistinen–Marburger (K-M)-type model [31,32]. Therefore, the same set of experiments enabled the determination of the E410NiMo theoretical martensite start temperature without applied stress, $T_{KM}$, and the transformation rate, $a_{m}$. These parameters are used in Equation (8) to determine the proportion of the martensite phase upon cooling.

(8) $$f_{\alpha^{\prime }}=1-e^{-a_{m}\left(T_{KM}-T\right)}$$

The parameters for both materials’ phase transformation models are listed in Table 3. However, in this study, the values of $T_{KM}$ refer to the previously defined Equations (4) and (5). The martensitic and austenitic phase coefficients of thermal expansion (CTE) of S41500 and E410NiMo are also listed in Table 3. These material parameters enable the modeling of the thermal and transformation strains associated with the proportion of the austenitic and martensitic phases and the transformation from one phase to another.

**Figure 9:**
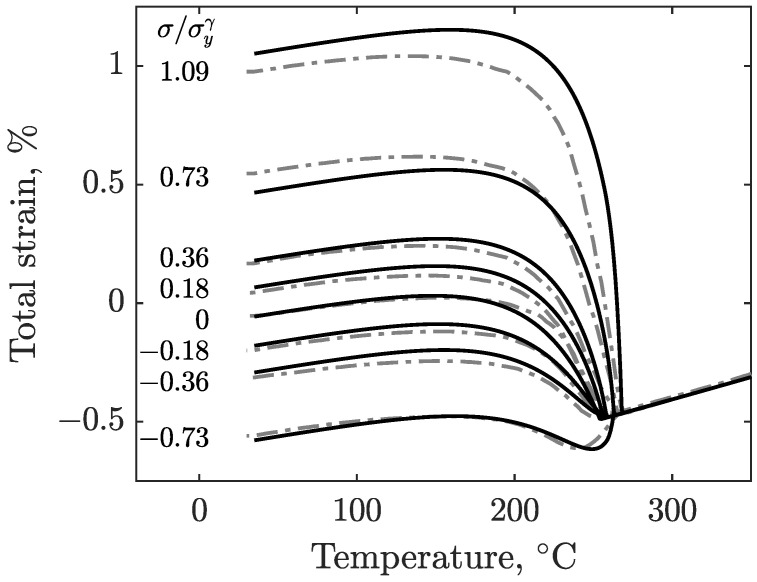
Comparison of modeled total strains (solid) and experimental total strains data (dashed) for S41500 stainless steel.

Figure 9 compares the modeled total strains to the strains measured during the transformation-induced plasticity experiments for S41500 stainless steel. Each of the strain rate components of Leblond’s model was computed using the previously defined parameters and by using an explicit scheme. A good agreement between the modeled behavior and experimental data was achieved. For stress ratios between −0.36 and 0.36, the maximum difference in the strain is 0.025 when room temperature is reached. For the higher stress ratio of 0.73, a maximum difference of 0.08% is recorded for the tensile test as the cooling ends. An additional experiment was conducted with a stress ratio of 1.09, exceeding the austenite yield strength. This last test makes it possible to visualize the behavior of the material when it undergoes a classical plastic strain component as the transformation occurs. For this test, the model overestimates the strains in the early transformation stage. A discrepancy is formed as the strains accumulate at a faster rate in the model. However, as the transformation continues, the gap gradually narrows and ends with a difference of 0.075% between the two strains.

**Figure 10:**
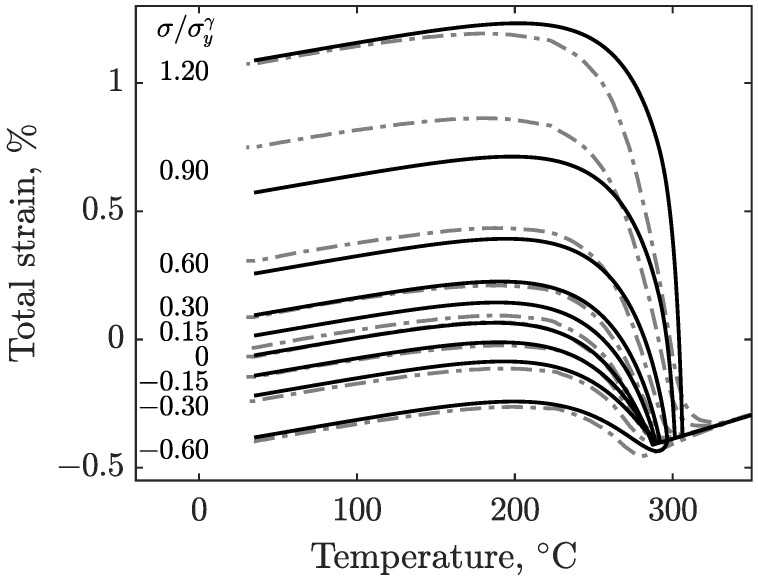
Comparison of modeled total strains (solid) and experimental total strains data (dashed) for E410NiMo stainless steel.

Figure 10 shows the results obtained using the model, along with the experimental values for the E410NiMo filler metal. Again, the model is in good agreement with the experimental data, especially for the lower stress ratios between −0.60 and 0.60, where the largest strain gap is 0.05% for the higher applied tensile stress ratio (0.60) test at room temperature. Two more tests with high tensile stress ratio levels were performed at 0.90 and 1.20 for the cases where the applied stress was close to or above the yield strength. A discrepancy is noticeable at the end of the 0.90 stress ratio test. For the 1.20 stress ratio, the model overestimates the strains at the start of martensitic transformation. However, as the cooling proceeds, the gap between the measured and modeled strains vanishes.

Feeding the model with the parameters presented here enables a consistent simulation of the material behavior during martensitic transformation under the effect of stresses for both materials. However, a discrepancy is observed for stress ratios close to or above the material yield strength. This disparity is due to different sources. One of these is the difficulty associated with measuring material properties for phases separately. In the present study, the austenite and martensite stress-strain curves were measured at $350{\;}^{{}^{\circ}}C$ to obtain a realistic elastic limit. However, these properties are used throughout the entire cooling process as this is the only temperature at which the material properties were evaluated. Additionally, although this temperature has been chosen to be close to $M_{s}$, it may not be high enough to completely avoid triggering the martensitic transformation at the beginning of plasticity. Moreover, the plasticity model considers a perfectly plastic material for both phases. This choice has been made since the TRIP coefficients are determined experimentally and, therefore, account mainly for the parent phase yield. Finally, in this study, the transformation parameter $\alpha_{m}$ of the K-M model was kept constant throughout the modeling of martensitic transformation. Recently, Liu et al. [33] reported better agreement with experiments when $\alpha_{m}$ is defined based on phase proportion and applied stress, especially for the early transformation steps.

## 4. Conclusions

In this paper, the material parameters required to model the transformation-induced plasticity of the 13Cr-4Ni base metal, as well as those of the filler material, during martensitic transformation were determined experimentally. For this purpose, the experiments were performed in a quenching dilatometer. The following conclusions can be drawn:

- An improvement to the measurement system was made, allowing for the use of specimen geometry that met the requirements of the tensile test standards.
- The TRIP coefficient’s *K* values for both S41500 steel and the E410NiMo filler material were determined as $8.12\times 10^{-5}$ and $7.11\times 10^{-5}$, respectively.
- The set of experiments was used to determine a linear relationship between applied stress and the martensitic start temperature ($M_{s}$) for both materials. This feature allows for martensitic transformation in the model to be triggered consistently with that in the experiments.
- The solid state phase transformation model parameters for the E410NiMo filler material were also determined following the same methodology used in the author’s previous work [25] for S41500.
- When fed with the material parameters found in this study, the Leblond model has been successfully compared with the experiments. The model showed very good agreement with the experiments and has proven effective in reproducing the transformation-induced plastic strain behavior of the stress levels used for TRIP coefficient determination. Although more discrepancy is observed at higher magnitudes of stress close to or beyond the austenitic phase yield strength, the model still provides results that are consistent with the experiments.

The presented work, thus, allows for the strains that occur during martensitic transformation in 13Cr-4Ni steels upon cooling with applied external loads to be predicted accurately. It also allows for the simulation of the manufacturing and repairing processes of hydraulic turbines, which will enhance residual stress calculations and fatigue life assessments.

## Figures and Tables

**Table 1:** Chemical compositions of UNS S41500 stainless steel and E410NiMo filler material.

Material	wt%	C	Mn	P	S	Si	Cr	Ni	Mo
ASTM	Std. [22]	<0.05	0.50–1.00	<0.030	<0.030	<0.60	11.5–14.0	3.5–5.5	0.50–1.00
S41500	Meas.	0.034	0.68	0.018	0.001	0.44	12.7	4.0	0.57
ASME	Std. [23]	<0.06	<1.00	<0.04	<0.03	<1.0	11.0–12.5	4.0–5.0	0.40–0.70
E410NiMo	Meas.	0.019	0.37	0.010	0.008	0.49	11.9	4.5	0.62

**Table 2:** Selected uniaxial loading ratio for the transformation-induced plasticity parameter determination experiments.

Material	Loading Ratio $\sigma /\sigma_{\gamma }^{y}$						
S41500	−0.73	−0.36	−0.18	0	0.18	0.36	0.73
E410NiMo	−0.60	−0.30	−0.15	0	0.15	0.30	0.60

**Table 3:** Material parameters for thermal strain calculations and phase transformation models.

Material	CTE, ${}^{{}^{\circ}}$C${}^{-1}$		JMAK, $\alpha^{\prime }\to \gamma$			K-M, $\gamma \to \alpha^{\prime }$	
	$\alpha^{\prime }$	γ	$E_{a},\frac{\mathrm{kJ}}{\mathrm{mol}}$	n	$K_{0}$	$a_{m}$, ${}^{{}^{\circ}}$C${}^{-1}$	$T_{\mathrm{KM}},{}^{{}^{\circ}}$C
S41500	2.2 $\times \;10^{7}T$ + 0.0011	7.0 $\times \;10^{7}T$ + 0.0017	509.9	0.66	4.04$e^{24}$	0.0267	Equation (4)
E410NiMo	3.5 $\times \;10^{7}T$ + 0.0011	7.4 $\times \;10^{7}T$ + 0.0016	468.9	0.58	7.31$e^{22}$	0.0280	Equation (5)

## Data Availability

Data are contained within the article.
